# ENT care of children and adolescents in the Brazilian public healthy system in three different municipalities

**DOI:** 10.1016/S1808-8694(15)30605-4

**Published:** 2015-10-18

**Authors:** Cheng T-Ping, Luc Louis Maurice Weckx

**Affiliations:** 1 MSc in otorhinolaryngology at the ENT and HNS Graduate Program at UNIFESP-EPM. PhD student at the ENT and HNS Graduate Program at UNIFESP-EPM. MD, ENT at Hospital João XXIII and Instituto de Otorrinolaringologia de Minas Gerais.; 2 Full Professor at the Department of Otorhinolaryngology and Head and Neck Surgery at UNIFESP-EPM. Head of the Graduate Program on Otorhinolaryngology and Head and Neck Surgery at UNIFESP-EPM. Pediatric Otorhinolaryngology Course at UNIFESP-EPM.

**Keywords:** adolescents, children, diagnosis, otorhinolaringology, treatment

## Abstract

The data base of ENT care in the Brazilian public health system (Sistema Unico de Saude - SUS) will help organize public health programs.

**Aim:**

The following items were investigated in patients aged up to 17 years attended in public health system outpatient units in the city of Mariana, in the ENT screening unit, UNIFESP-EPM, and in CISMISEL: 1) The main otorhinolaryngological diagnoses; 2) The most frequently required exams, drugs, and surgical procedures and their indications; 3) The jobs of parents; the number of siblings; and 4) A statistical analysis and comparison of data in each location.

**Material and Method:**

We undertook a prospective study and a statistical analysis of variables that were gathered during the first visit.

**Results:**

The age, the parents’ salary, the number of siblings aged below 18 years, the presence of rhinitis, ears diseases, the exams, drugs and otological surgeries that were indicated were all statistically significant.

**Conclusion:**

The most common diagnosis was mouth breathing. The most common surgery was adenotonsillectomy. The most frequently requested exam was a lateral cranial radiograph. The number of unemployed parents, their poor salaries, and the number of siblings make if difficult for these patients to be treated in any facility other than the public heath system.

## INTRODUCTION

Very little information is available on the otorhinolaryngology services provided by the SUS (Brazilian Public Health Care System). Lack of information on things such as most prevalent diseases in ENT practice, ordered complementary tests, clinical and surgical treatment, and patient family income levels hamper the development of preventive and curative health care policies. The development of health care information systems has been enacted in article 190-XV of the Minas Gerais State Constitution1 whereas the epidemiology-based action plans are enacted in articles 5 and 6 from Federal Law 80802-4. There is still a lot to be done to comply with these legal requirements, as seen from the situations encountered by SUS ENT patients.

The age of 17 was picked as the upper limit for our study, as in many ways these patients still depend on the support provided by their parents or caretakers, i.e., they are not fully legally autonomous to resolve every aspect of their daily lives. If their parents’ income is too low or if they are unemployed, scarcely ever will they be able to pay for complementary tests or buy medication (corticosteroids, antihistaminic drugs, and antibiotics) unless these are available at no charge from the public health care system.

Delay in reaching a diagnosis and treating ordinary diseases in the ENT realm such as otitis, rhinosinusitis, and mouth breathing among others, may predispose patients to chronic conditions, complications, and sequelae that will eventually require more complex and expensive treatment^5^, ^6^, ^7^, ^8^, ^9^.

The development of a database on the ENT services offered by the SUS may provide for valuable information to the public health care system, government agencies, non-governmental organizations, health care professionals, and society as a whole on the priorities upon which ENT care should be based, such as most prevalent diseases, most frequently ordered complementary tests, most commonly prescribed drugs and most indicated surgical procedures. Such information enables the organization of strategies to tackle collective health problems, optimize expenditure, and justify the creation of new funding options, both public and private.

## OBJECTIVE

Define for patients up to 17 years of age:

1) the main diagnosed ENT diseases;
2) the most frequently ordered complementary tests;
3) the most commonly prescribed drugs;
4) the most frequently conducted outpatient procedures and surgical indications;
5) parental labor status and number of siblings living in the household;
6) compare and statistically analyze the data collected for the three studied municipalities.

## MATERIALS AND METHOD

Only the data gathered during the patients’ first appointment with the physician was included. All data was prospectively collected and only patients of up to 17 years of age accompanied by their parents or caretakers that looked for ENT care at the public clinic in Mariana, at the CISMISEL (Consórcio Intermunicipal de Saúde da Microrregião de Sete Lagoas - Inter-municipal Health Care Consortium for the Sete Lagoas Micro-region) headquarters, and at the ENT screening service of the Universidade Federal de São Paulo (UNIFESP) - Escola Paulista de Medicina (EPM). All patients were seen by one same researcher from each given facility.

Age, complaints, ordered complementary tests, diagnosis, prescribed medication, surgical indication, number of under-17 siblings, and parental labor status were already included in the patients’ records. This is an observational, prospective, and cross-sectional study. Category variables were studied using Cramèr's V Ratio and numeric variables were analyzed through fixed -value Variance Analysis.

Patients were informed of the research and were seen regardless of their willingness to join in, and were treated and followed based on their clinical needs. The Research Ethics Committee at UNIFESP - EPM analyzed and approved this project under permit 0056/03.

The municipality of Mariana is located in Minas Gerais (MG), and has an estimated 46,710 inhabitants as per the 2000 Census. The Policlínica de Mariana is a municipal outpatient ward connected to the SUS. ENT service is provided in the form of elective visits and urgencies referred by the municipal health care network. Complementary tests available at the Policlínica are x-rays and a few biochemical and hematologic tests. ENT-specific complementary tests are conducted at private clinics in Mariana, Ouro Preto or Belo Horizonte and are paid for by the patients or by the municipal authorities. Other drugs often available for distribution by the public health care system are analgesics (dipyrone, paracetamol), anti-inflammatory drugs (diclofenac), oral corticosteroids (prednisone), antibiotics (amoxicillin, ampicillin, cefalexin, trimethoprim-sulfamethoxazole, and erythromycin), anti-acids (ranitidine, cimetidine) and antivertigenous drugs (cinnarizine). All medication that is not publicly available is paid for by the patient or bought with the aid of municipal authorities. Surgery patients are referred to Belo Horizonte.

The ENT screening service at UNIFESP-EPM sees patients sent from other medical services at Hospital São Paulo, UNIFESP-EPM and those coming from health care centers under Regional Health Care Center 5 (Núcleo Regional de Saúde 5 - NRS-5). The NRS-5 covers 21 districts and an estimated at 2,414,868 people as per the 1999 survey done by the Metropolitan Regulation System (Sistema de Regulamentação Metropolitano - SRM) under the State Secretary of Health. ENT complementary tests are offered by the Department of Otorhinolaryngology, and Head and Neck Surgery. Prescribed medication is not offered free of charge. Surgery candidates and complex cases are referred to each specific branch within the Department of Otorhinolaryngology.

The CISMISEL (Consórcio Intermunicipal de Saúde da Microrregião de Sete Lagoas - Inter-municipal Health Care Consortium for the Sete Lagoas Micro-region) covers the municipalities of Araçaí, Cachoeira da Prata, Caetanópolis, Cordisburgo, Fortuna de Minas, Inhaúma, Jequitibá, Paraopeba, Santana do Pirapama and Sete Lagoas in Minas Gerais, and serves a total of 249,161 people (2000 IBGE Census). Except for Sete Lagoas, where an otorhinolaryngologist is available in the municipal health care system, none of the municipalities has ENT care available locally. Each town has a monthly quota of visits proportional to their contribution to the system. All complementary ENT tests ordered within the CISMISEL are forwarded to their towns of origin. They first attempt to resolve the matter locally. If all fails, the order is referred to Sete Lagoas or Belo Horizonte. Drugs are paid for with the aid of the municipality or by the patient alone. Except for Sete Lagoas, where some surgical procedures are carried out, none of the towns can perform surgery locally and all patients are referred to Belo Horizonte.

## RESULTS

Data from 435 first-time visits were collected at Policlínica de Mariana, 410 at ORL UNIFESP-EPM, and 316 at CISMISEL. In all, data from 1,161 patients was collected - 645 males and 516 females. Considering Cramèr's V Ratio and a 5% significance level, there was no statistically significant difference in relation to gender among the sites included in this study. Average ages were of 7.7 years at Policlínica, 5.9 at Triagem, and 8.2 at CISMISEL. Looking again at the 5% significance level, age behaved similarly at Policlínica and CISMISEL, whereas at Triagem the average age was lower.

**Chart 1:**
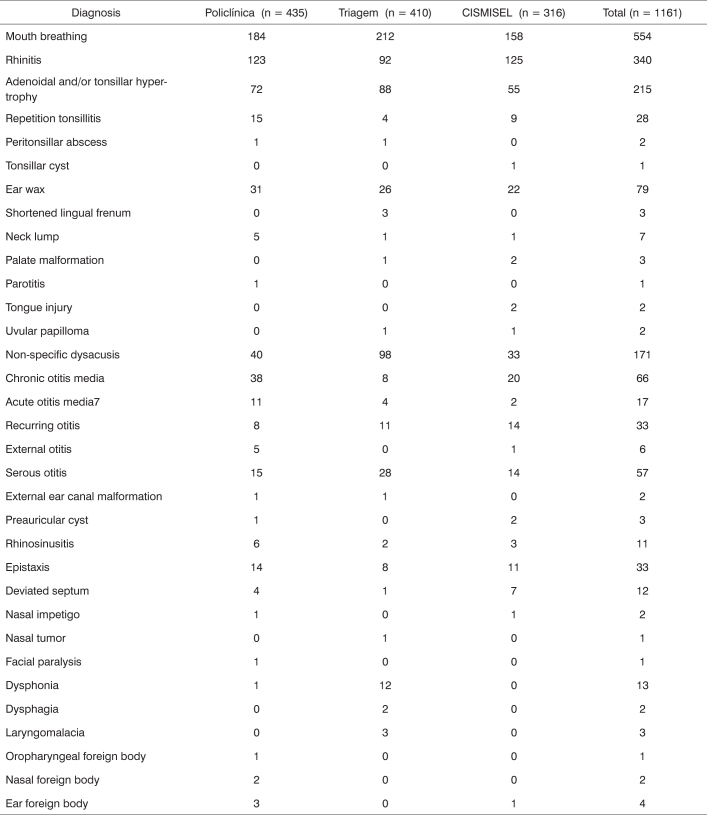
Clinical diagnosis during first ENT visit.

From the 1,161 patients captured in our sample (Chart 1), 554 are mouth breathers (47.7%) - the most frequently found diagnosis, regardless of etiology, be it in general or at each of the researched sites. Rhinitis was diagnosed in 340 patients (29.9%) and adenoidal and/or tonsillar hypertrophy was found in 215 patients (18.5%).

From the 435 patients seen at Policlínica de Mariana, mouth breathing was found in 184 patients (42.3%). Non-specific rhinitis was diagnosed in 123 patients (28.3%) and adenoidal and/or tonsillar hypertrophy in 72 patients (16.6%). From the 410 patients seen at Triagem, mouth breathing was observed in 212 patients (51.7%), rhinitis in 92 (22.4%), and adenoidal and/or tonsillar hypertrophy in 88 (21.5%). From the 316 patients seen at CISMISEL, mouth breathing was identified in 158 patients (50%), rhinitis in 125 (39.6%), and adenoidal and/or tonsillar hypertrophy in 88 (17.4%).

Non-specific dysacusis, without apparent relation to otitis, ear malformation, or excessive ear wax, was left to be diagnosed after complementary tests were done and was found in 171 patients (14.7%). Seventy-nine patients (6.8%) came to see the physician due to excessive ear wax, and in general improved their hearing after wax removal. Wax removal was done in 86 of the 1,161 patients (7.4%).

Otitis media (Chart 1), in its various manifestations, was present in 171 patients (14.7%), 68 (5.8%) of whom had chronic otitis media and 57 (4.9%) had serous otitis media.

**Chart 2:**
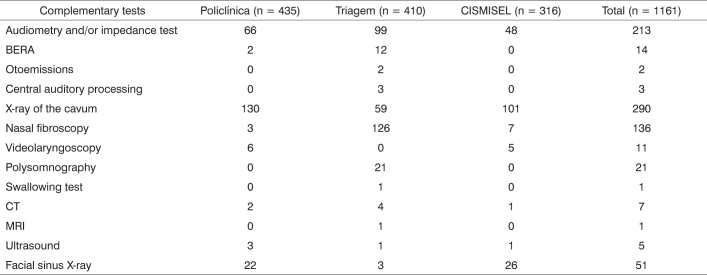
Ordered complementary tests.

**Chart 3:**
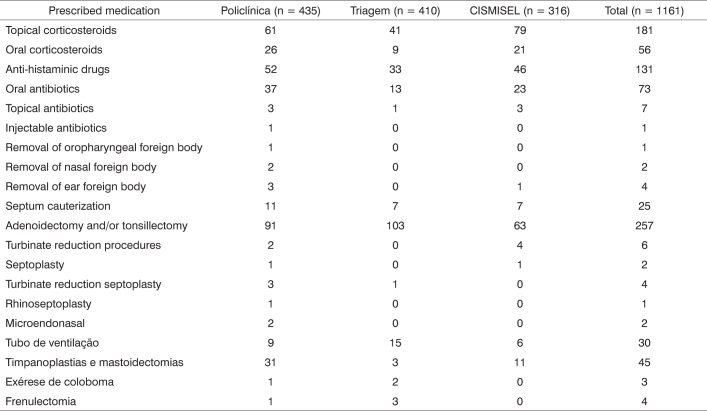
Prescribed medication, performed procedures, and indicated surgery.

X-rays of the cavum were done in 290 patients (25%), audiometry in 213 (18.3%) and nasal fibroscopy in 136 patients (11.7%) (Chart 2). Topical corticosteroids and oral anti-histaminic drugs were the most frequently prescribed medications, and were indicated to 181 (15.6%) and 131 (11.3%) patients respectively (Chart 3). The most commonly indicated surgical procedures were adenotonsillectomy and tympanoplasty and mastoidectomy, with 257 (22.1%) and 45 (3.9%) patients respectively (Chart 3).

When looking at labor status for the parents of the 1,161 patients included in our sample, 577 fathers (49.7%) and 829 mothers (71.4%) were unemployed. 109 (9.4%), 111 (9.7%), and 54 (4.7%) patients at Policlínica, Triagem, and CISMISEL were only children. The average of siblings of up to 17 years of age for patients who were not only children was of 3.408 at Policlínica, 2.003 at Triagem, and 2.336 at CISMISEL. Considering a 5% statistical significance level, variable ’labor status’ behaves similarly at Policlínica and CISMISEL and differently at Triagem, where fewer parents are unemployed; variable ’number of siblings of up to 17 years of age’ behaves similarly at Policlínica and CISMISEL and differently at Triagem, where patients had fewer siblings on average.

**Chart 4:**
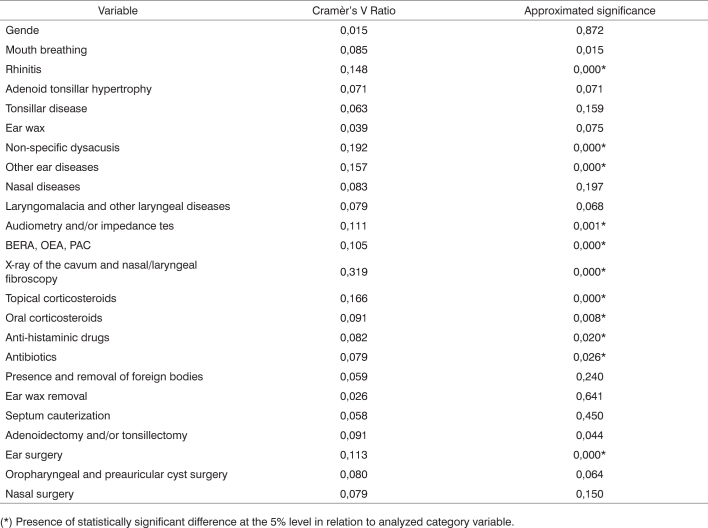
Comparison between category variables of all three institutions through Cramèr's V Ratio.

Chart 4 reveals a statistically significant differences in category variables ’rhinitis’, ’non-specific dysacusis’ and ’other ear diseases’ (rhinitis was more frequent at CISMISEL, dysacusis at Triagem and other ear diseases at Policlínica); ’complementary tests’ (hearing tests and nasal fibroscopy were available at Triagem, but not at Policlínica and CISMISEL; X-ray of the cavum was more present at these two sites as it was the most promptly available method to assess adenoids); ’prescribed medication’ (Triagem prescribed all sorts of medication less frequently and at CISMISEL there were more prescriptions of topical corticosteroids), ’ear surgery’ (left ear tympanoplasty was more indicated, more specifically at Policlínica, and ventilation tubes were more prevalent at Triagem).

(*) Presence of statistically significant difference at the 5% level in relation to analyzed category variable.

## DISCUSSION

Not always is it possible to reach a diagnosis for the patient in his/her first visit to the physician. Patient complaints are written down and from them hypotheses are drawn, and they may or not be confirmed through complementary tests and during follow-up. Had this study not been limited to looking only at the patients’ first visit, many complaints would have been turned into diagnoses during the subsequent follow-up period.

The Pediatric Otorhinolaryngology Course at UNIFESP-EPM started the first Center for Mouth Breathing in Brazil in 2002, in a pioneering effort to offer multidisciplinary treatment (allergy care, physical therapy, speech and hearing therapy, dental care, and ENT) to this condition. The center was opened as elevated prevalence rates of this condition - whose resolution is rarely satisfactory when only one area of medical expertise is involved - began to be observed in the daily ENT care provided at the institution. One of the goals of the center is to offer patients all specialized care they need within one same day, thus reducing patient expenditure with transportation and mitigating treatment discontinuation. Aside from health care, the center also conducts research on mouth breathing using the expertise of the various medical areas present there, generating information that may aid in offering increasingly better service to patients. Article 46 in Law 8080 defines that the SUS (Brazilian Public Health Care System) must promote the development and sharing of technology and knowledge throughout the entire health care chain. In this sense, partnerships between the public and private sectors in the areas of service and research - as is the case for the Center for Mouth Breathing - make it possible for SUS patients to be properly served and for health care professionals to be aware of the findings resulting from research work.

The sheer number of mouth breathers found at Policlínica de Mariana and CISMISEL call for the creation of a center to serve local patients using a multidisciplinary approach and equipped with the ability to provide all complementary tests required to identify the causes to the condition, as defined in Article 188 of the State Constitution^1^. Multidisciplinary care is however expensive, as many are the required areas of medical expertise, complementary tests, and cases that will require dental and surgical treatment. Timely and proper treatment can certainly prevent complications and sequelae from occurring^9^. The Brazilian Society of Otorhinolaryngology and Head and Neck Surgery, as one of its assignments, ought to divulge the importance of offering proper treatment to mouth breathers. The elevated prevalence rates of mouth breathing found in such disparate towns call for more investments by the public authorities in the hiring of human resources and construction of facilities and in the setting up of partnerships with the private sector and non-governmental organizations.

Rhinitis is a condition that involves a significant portion of the population. Nasal obstruction, itchy nose, nasal discharge, hyposmia, itchy eyes, and teary eyes when triggered through contact with certain substances or environmental and climatic conditions, suggest the presence of allergic rhinitis. However, differential diagnosis for rhinitis could not be done as both Prick-test and RAST are not readily available at the SUS sites in these towns. Statistically significant differences were found in the prevalence rates of rhinitis in the three towns. More cases were found at CISMISEL, for no apparent reason. Rhinitis prevalence rates found in the sample and at each town matches the findings described by Lundback et al. ^10^ and Settipane et al.^11^. The high cost of topical corticosteroids, second generation oral anti-histaminic drugs, and specific immunotherapy - the latter not available at the public health care system - associated with economic hardship, end up impairing or even rendering impossible any environmental control measures and drug therapies. For mouth breathers, treating adenoid and tonsillar hypertrophy together with rhinitis is fundamental for both dental and speech and hearing therapy approaches.

Dysacusis in the early stages of life leads to impairments in speaking and learning skills in general. Sensorineural causes, although not accompanied by differential diagnosis, appeared less frequently than conductive ones, and will possibly require the patients to wear hearing aids and go to speech and hearing therapists. Conductive causes such as serous otitis media may adversely affect speech development and evolve in the long run, thus requiring careful diagnosis and adequate treatment^5^, ^6^, ^8^, ^12^, ^13^. In both cases early identification and treatment are essential to providing patients with good quality-of-life levels as set in Article 190 of the State Constitution1. The equipment needed to assess hearing loss is quite expensive and requires trained personnel. Neither Policlínica nor CISMISEL have such equipment. The number of complaints derived from dysacusis and the prevalence of chronic otitis in these towns call for actions to facilitate hearing loss assessment, whether it is through public acquisition of audiometers, impedance test devices and acoustically insulated booths, or partnerships with private health care institutions. The solution, however, does not lie exclusively in offering tests to patients. It is also important to develop facilities equipped with otorhinolaryngologists, speech and hearing therapists, and mental health care professionals working in a coordinated fashion while approaching patients with dysacusis, as defined in Article 6 of Law 8080. At Triagem, all necessary tests are available. Equipment, speech and hearing therapists, and graduate research projects connected to the courses on Otology, Otoneurology, and Pediatric Otorhinolaryngology make it possible for complex tests, therapy, and follow-up to be offered. However, as it is a reference center serving a multitude of patients, delays may occur. It is important for hearing loss patients to have a chance to learn to communicate, read, and write so they can be better integrated into society and live off of their jobs, without having to apply to pensions from the public welfare system.

The relevance of offering certain complementary tests locally depends on how prevalent the diseases targeted by the tests are, on the tests’ diagnostic value, and on how they impact the course of treatment. Highly prevalent diseases justify test equipment acquisition or partnering up with local private health care institutions. Complementary tests for less prevalent diseases in towns of smaller populations can be ordered at larger centers, and do not call for the acquisition of expensive equipment that would only occasionally be utilized. Articles 197, 198, and 199 from the Federal Constitution open the possibility for private health care institutions to participate in public health care, as long as such participation is managed by each State individually.

Considering the number of unemployed parents and the low income levels of those who have a job, the difficulties in offering medication at no charge for patients, the scarcely available complementary tests, and the few surgical procedures available at Policlínica and CISMISEL, alongside the patient-crowded Triagem at ORL UNIFESP-EPM (leading to delays in performing tests and surgical procedures), the more children in the household with up to 17 years of age, the more stressed will be the family budget, making it difficult or even impossible for parents to afford clinical or surgical treatment outside the scope of the SUS.

Statistically significant differences were found for some of the variables due to characteristic differences between the sites. These differences, however, do not preclude the development of similar prevention and treatment projects to address mouth breathing and ear diseases in all three towns. Although the Federal and State Constitutions and the SUS regulations ensure the right to comprehensive and free-of-charge health care and treatment (Article 196 from the Federal Constitution), the daily operation of the institutions analyzed in this study reveals shortcomings in various aspects of the SUS ENT care they provide.

The Constitution often puts the responsibility over health care on the shoulders of the State, but does not completely relieve society from participating and setting measures to achieve social improvement (Law 8069, Article 4; Minas Gerais State Constitution Article 186-IV). Health care professionals, whether from the public or private sector, would thus have an important role in promoting health care in general. Nonetheless, attempts aimed at improving local operational conditions, or even more comprehensive health-related action plans, must first consider the main demands from the population to be benefitted, whether or not they are to be funded publicly.

## CONCLUSION

The data gathered from the 1,161 first-visit patients, further divided into three groups of 435, 410 and 316 patients belonging respectively to Policlínica de Mariana, Triagem ORL UNIFESP-EPM and CISMISEL, was analyzed using Cramèr's V Ratio and fixed-value Variance Analysis and allowed the following conclusions:

1. Mouth breathing was the most commonly made diagnosis, with rhinitis and adenoid and tonsillar hypertrophy as the main causes; next are non-specific dysacusis, ear wax, chronic otitis media, and serous otitis media.
2. The most frequently ordered complementary tests were cavum X-rays, followed by audiometry/impedance tests and nasal fibroscopy, in agreement with the most frequent diagnoses.
3. Endonasal topical corticosteroids were the most prescribed drugs, followed by oral anti-histaminic drugs, oral antibiotics, and oral corticosteroids.
4. Adenotonsillectomy was the most frequently indicated surgical procedure, followed by tympanoplasty, mastoidectomy, and ventilation tubes, in agreement with the most frequent diagnoses.
5. Approximately half of the parents of the sampled population were unemployed, i.e., unemployment rates are alarming among SUS patients. Family income levels are quite low. Unemployment, low salaries, and number of children in the household have a severe impact on the families’ ability to afford decent health care.
6. Statistical analysis revealed the differences between variables age, labor status, and number of siblings of up to 17 years of age, rhinitis, non-specific dysacusis, and other ear diseases, ordered complementary tests, prescribed medication, and ear surgery indications in the three towns studied. Despite the differences, the economic hardship experienced by public health care patients must be considered whenever public health care strategies are developed.
